# Characterization of Oral Immunity in Cases and Close Household Contacts Exposed to Andes Orthohantavirus (ANDV)

**DOI:** 10.3389/fcimb.2020.557273

**Published:** 2020-11-03

**Authors:** Constanza Martinez-Valdebenito, Camila Andaur, Jenniffer Angulo, Carolina Henriquez, Marcela Ferrés, Nicole Le Corre

**Affiliations:** ^1^Departamento de Enfermedades Infecciosas e Inmunología Pediátricas, División de Pediatría, Escuela de Medicina, Pontificia Universidad Católica de Chile, Santiago, Chile; ^2^Laboratorio de Infectología y Virología Molecular, Red Salud UC Christus, Santiago, Chile; ^3^Laboratorio de Virología Molecular, Instituto Milenio de Inmunología e Inmunoterapia (IMII), Santiago, Chile

**Keywords:** Hantavirus Andes, innate immunity, saliva, orthohantavirus, mucins

## Abstract

**Background:** Andes orthohantavirus (ANDV) is the sole etiologic agent of Hantavirus Cardiopulmonary Syndrome in Chile and, until now, the only Hantavirus known to be transmitted by person-to-person route. The main risk of person-to-person transmission is to be a sexual partner of an index case, and deep kissing the main mechanism of infection. Experimental reports suggest that ANDV infection can be inhibited by some saliva components. Therefore, some host factors like saliva quality, could help to explain why some individuals do not become infected even though their exposure to the virus is high.

**Aim:** To compare some saliva components, such cytokines and mucins, between ANDV-infected cases (exposed-sick), their close household contacts (exposed-not sick) and healthy control not exposed.

**Methods:** Sixty-nine confirmed ANDV-infected cases, 76 close household contacts exposed to ANDV but not infected (CHC) and 39 healthy control not exposed (HCNE). The following components were measured in saliva: secretory immunoglobulin A (sIgA) by ELISA; cytokines (IL1β, IL12p70, TNFα, INFy, IL10, IL6, VEGF, IP10, and IL8) by Multiplex Assay and mucins MUC7 and MUC5B by Western Blotting.

**Results:** Among infected cases, CHC and HCNE analyzed 74, 45, and 33% were men, respectively (*p* ≤ 0.05). The average age for cases, CHC and HCNE was 37.7, 28.7, and 32 years, respectively (*p* ≤ 0.05). The average concentration of sIgA in infected cases was 4.846 mg/mL, higher than for CHC group, 0.333 mg/mL (*p* ≤ 0.05). For cytokines, significant differences were found comparing all groups for IFNy, IL12p70, and IL8. Among CHC group, there was a higher frequency of detection of MUC7 isoform (62.6%; 31/49) compared to ANDV-infected cases (40.5%; 17/42) (*p* < 0.05). Similarly, presence of MUC5B was higher among CHC groups (62.16%; 46/74) than in ANDV-infected cases (44.4%; 28/63) (*p* < 0.05).

**Conclusions:** Three salivary components showed differences between infected cases and close household contacts (sIgA, cytokines, and mucins). These differences can be explained by the acute state of the disease in the ANDV-infected cases group. However, the differences in MUC5B and isoforms of MUC7 are not entirely explainable by the infection itself. This work represents a novel description of salivary components in the context of ANDV infection.

## Introduction

The Andes Orthohantavirus is a member of the *Hantaviridae* family, *Orthohantavirus* genus (ANDV), and is the sole etiologic agent of the Hantavirus Cardiopulmonary Syndrome (HCPS) in Chile and the south of Argentina. This zoonotic virus has as main reservoir the *Oligoryzomys longicaudatus* (Toro et al., 1998; Medina et al., 2009; Figueiredo et al., 2014) and human represents an accidental host (Schmaljohn and Hjelle, 1997; Fields et al., 2013). The ANDV is mainly acquired through the inhalation route, from the environment contaminated with rodent fluids containing the virus. Other routes of contagion have been proposed based on the epidemiological background of the cases, such as direct inoculation by mouse bite, or the gastrointestinal route after eating food contaminated with the virus (Ferres et al., 2020). The ANDV is the only hantavirus, until now, that is transmitted from person to person, although in a low proportion of cases. In this situation, the contaminated fluids of the oral cavity, respiratory tract, blood, breast milk, and urine are potential vehicles of viral transmission (Mertz et al., 2006; Ferres et al., 2007; Martinez et al., 2010; Martinez-Valdebenito et al., 2011).

Regarding ANDV infection, acquired either through environmental or human to human exposure, one of the questions that still remains unclear is why if one or more subjects are exposed to the same risk factor, only one or two of them become infected. Saliva and oral secretions represent a natural barrier for trapping microbial agents trying to enter the human body. Saliva has an immune function that is related to mucosal-associated lymphoid tissue (MALT). One of the components of this function is secretory immunoglobulins (sIg), and in particular salivary IgA (slgA), produced by plasma cells as an adaptive immune response to specific antigens (Scannapieco, 1994; Marsh et al., 2009). In Puumala (PUUV) infection, a European hantavirus responsible for the epidemic nephropathy, specific sIgA was detected observing an inverse correlation with the viral RNA present in this same fluid (Pettersson et al., 2011). In human immunodeficiency virus (HIV) infection, it was observed that mucosal IgA antibodies in exposed and seronegative individuals have antiviral activity against HIV (Devito et al., 2000).

Some other proteins present in saliva have important antiviral properties, such as lactoferrins, histatin 5, lysozyme, and mucins. In *ex-vivo* experiments, it was demonstrated that the infectivity of the Hantaan virus (HTNV) was only inhibited by mucins (Hardestam et al., 2008). Interestingly, the propagation of ANDV in presence of saliva was inhibited in a smaller proportion in *ex-vivo* experiments with PUUV and HTNV (Hardestam et al., 2009) suggesting that ANDV is more resistant to this oral fluid and mucosal barrier. The mucins contained in saliva are considered as important components of the innate immune response due to their ability to bind and agglutinate bacteria (Segal and Wong, 2008; Hardestam et al., 2009). Mucins are divided into two large groups considering their structure and function: a high molecular weight MUC5B (MG1), which protects against chemical, physical and microbial damage; and MUC7 (MG2) whose role is focused on oral cleansing of bacteria (Segal and Wong, 2008).

Based on this background, we hypothesize that there could be differences in the composition of oral components, such as sIgA, cytokines, and mucins in subjects who were successfully infected with ANDV compared to subjects exposed to the same risk factor but did not become ill. In addition to enriching the knowledge of immunity to ANDV infection, these differences may contribute to a better understanding of individual susceptibility to the development or inhibition of infection. The aim of this study was to compare the composition at the level of proteins and salivary components of ANDV-confirmed cases (exposed-patients) and their close household contacts who did not develop the infection (exposed-not-sick).

## Methods

### Study Population

- **Cases:** Samples of 69 ANDV-infected cases were analyzed (index cases). Cases were confirmed through positive IgM serology specific for ANDV and/or by the specific detection of viral RNA through reverse transcription and quantitative polymerase chain reaction (RT-qPCR) (Padula et al., 2000; Vial et al., 2016).
- **Close household contacts:** Samples from 76 close household contacts exposed to sick cases or to common environmental risk factors but who did not get infected after 5 weeks of follow-up were analyzed. These close household contacts slept in the same bed or had close contact with asymptomatic ANDV case during 30 days before or 7 days after the onset of symptoms.
- **Healthy controls not exposed:** Saliva samples were obtained from 39 healthy subjects without documented ANDV infection, paired with the other two groups by sex and age. None of these subjects had a history of periodontal disease.

Participants from cases and close household contacts groups were enrolled during the period 2008–2017, meanwhile healthy controls not exposed were enrolled during 2019–2020. In addition, epidemiological and demographic data were collected through a previously validated questionnaire (Ferres et al., 2007).

#### Ethical Statement

Ethical approval for the use of samples, data and protocol design was approved by the Ethics Committee of the Faculty of Medicine. Pontificia Universidad Católica de Chile (Code 12-292 and 16-092). All participants signed an informed consent at the time of enrollment, this consent was approved by the same committee.

### Sampling

- **Saliva**: The time of sampling collection from the cases and close household contacts, corresponded to the day of hospital admission and or the day on which the diagnosis of Hantavirus disease was made for the case (visit 1). Then on day 60 a new sample was obtained (60 +/– 7 days), and all the subjects were asymptomatic. The third group, healthy controls, gave a single saliva sample. To obtain the saliva sample, the participant was asked to refrain from eating or brushing their teeth for at least 30 min prior to taking the sample. The Salivette® cotton was placed under the tongue for 30 s, then removed and placed within the collecting tube and processed within 24 h. The processing involved centrifuging the cotton inside the collecting tube to squeeze the saliva toward the tube's lower chamber. The aliquots were frozen at −80°C, until processing. These samples were thawed at room temperature (RT) for processing.
- **Serum**: Serum from ANDV-infected patients were obtained on the day of hospital admission and stored at −80°C until processing. For processing, serum samples were thawed at RT.

#### Quantification of Total Proteins in Saliva

For the processing and measurement of the salivary proteins, the quantification of total proteins was initially carried out using the Bradford colorimetric method, using a commercial kit (Bio-Rad Protein assay. cat: 5000001). A standard curve was developed using different concentrations of Bovine Serum Albumin (BSA) (0–8 mg/mL). Using a linear regression equation, the total protein concentration in mg/mL was calculated for all samples.

#### Quantification of sIgA

For the measurement of sIgA, a specific commercial ELISA system (Salivary secretory IgA elisa kit. # 1-1602. salimetric) was used following the manufacturer's instructions.

#### Quantification of Cytokines

For the cytokines quantification, the multiplex detection system was used, operating a kit designed for the measurement of the following proteins: IL1β, IL6, IL8, IL10, IL12p70, IP10, IFNy, TNFα, and VEGF (HCYTOMAG-60K, Millipore, Merck). The procedure was performed as specified by the manufacturer and made on the MAGPIX Luminex® device.

#### Detection of MUC7 and MUC5B

Mucins MUC7 and MUC5B were detected by Western Blotting. Briefly, 10% SDS-PAGE was performed for MUC7 and 6% for MUC5B. In each gel, a maximum of 20 μg of total protein was loaded per well. The SDS-PAGE gels were resolved at 100 V for 1.5 h, and transferred to a nitrocellulose membrane (100 V for 1 h for MUC7, 60 V for 15 h in a cold room for MUC5B). Membranes were blocked with TBS containing 5% skimmed milk and 0.1% Tween 20 for 1 h at room temperature, washed three times with TBS containing 0.1% Tween 20, and incubated overnight with the primary antibody. The primary anti-MUC7 antibodies (abcam cat: 105466) and anti MUC5B (abcam cat: 105460) or EU-MUC5B (kindly provided by Prof. Julieta Gonzalez, Universidad de Chile) were incubated at 1:1,000 dilution. Membranes were then washed three times and incubated for 1 h with the rabbit anti-mouse IgG secondary antibody, conjugated with peroxidase (abcam cat: 97046) at 1:5,000 dilution. The detection of proteins was visualized by enhanced luminescence using a chemiluminescence reaction (Pierce ECL Plus Western Blotting Substrate, cat: 32132. Thermofisher®) according to the manufacturer's instructions.

#### Statistical Analysis

Statistical analyzes were carried out with the statistical program GraphPad Prism 6, using the Mann Whitney test and Kruskal-Wallis test for unpaired variables and Wilcoxon rank test for paired variables, with a significance level of *p* < 0.05. For categorical variables, the chi-square test was used with a level of significance of *p* < 0.05.

## Results

### Demographic Description of the Study Population

The demographic characteristics are described in Table 1. ANDV-infected individuals were predominantly male (74%), with an average age of 37.7 years. Meanwhile, close household contacts and healthy controls not exposed were most frequently women (55 and 67%, respectively), with an average age of 28.7 and 32 years, respectively. These characteristics were significantly different between the groups. On the other hand, Hispanic ethnicity was the predominant ethnic group in all three groups.

**Table 1:** Demographic description of 69 cases, 76 close household contacts and 39 healthy controls not exposed.

	**ANDV-infected cases *n* (%)**	**Close household contacts *n* (%)**	**Healthy not exposed *n* (%)**	*p-*value^*^
Age in years, mean [range]	37.7 [0.75–79]	28.7 [0.33–71]	32 [7–66]	**0.016^a^**
Sex, men	51 (74)	34 (45)	13 (33)	**<0.0001**
Ethnicity				
Hispanic	53 (77)	50 (66)	39 (100)	
Native	6 (9)	7 (9)	0	0.058
Other	8 (12)	7 (9)	0	

* *Chi-square test and Kruskal-Wallis test <0.05 = Significant*.

a *Significant only for ANDV-infected cases and Close household contact*.

### Description of Cytokines and sIgA in Saliva of Cases, Close Household Contacts, and Healthy Controls Not Exposed

**Figure 1:**
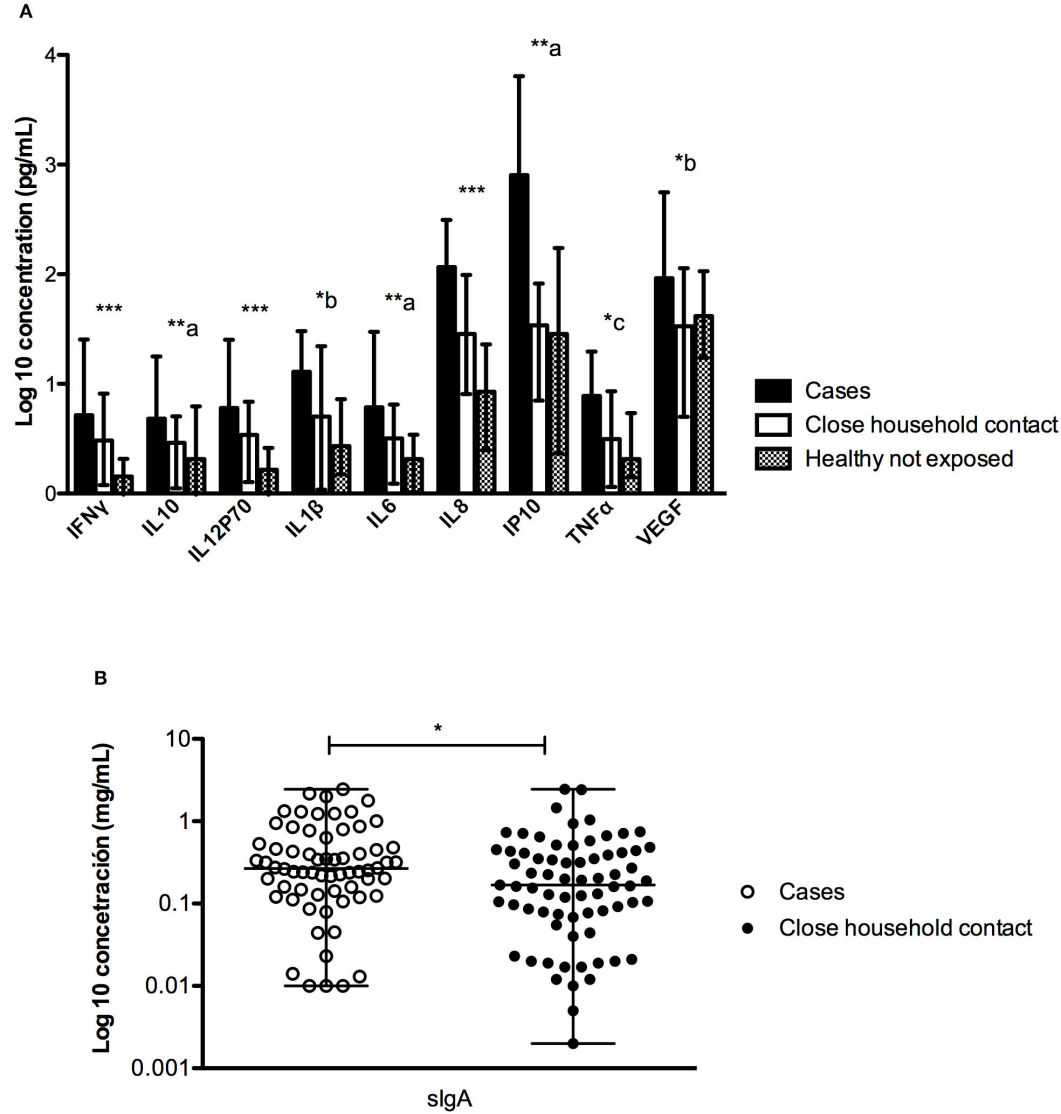
Comparison of the concentration (pg/mL) of different factors measured in saliva between ANDV-infected cases, close household contacts and healthy controls not exposed. **(A)** Quantification (pg/mL) of INFγ, IL10, IL12p70, IL1β, IL6, IL8, IP10, TNFα, and VEGF in all groups; dark boxes represent ANDV-infected cases, clear boxes represent close household contacts, and gray boxes represent healthy controls not exposed; Boxes represent median values and bars interquartile range. Comparisons are significance between columns: ***All group are statistically significant, **a: all group are statistically significant, except for Healthy controls not exposed and Close household contact, *b: only between Cases and Healthy controls not exposed is statistically significant, *c: only between Cases and Close household contact is statistically significant (Kruskal-Wallis test and Mann Whitney test, *p* < 0.05 = significant). **(B)** Quantification (mg/mL) of sIgA in first two groups; clear circles represent ANDV-infected case and dark circles represent close household contacts; whiskers represent minimum and maximum values and horizontal bar in the box the median value. *p < 0.05 (Mann Whitney test).

The concentration of different immune factors present in the saliva of three groups was compared. As shown in Figure 1 and Supplementary Table 1, significant differences were found for all the immune factors analyzed. The 9 cytokines were significantly higher in ANDV-infected individuals compared to the other groups. Interestingly, we found statistical differences between healthy control not exposed and close household contact, particularly in INFγ, IL12p70, and IL8 (Figure 1A). Regarding sIgA, it was possible to measure it only in cases and their close household contacts, observing a significantly higher concentration in ANDV-infected patients (Figure 1B).

**Figure 2:**
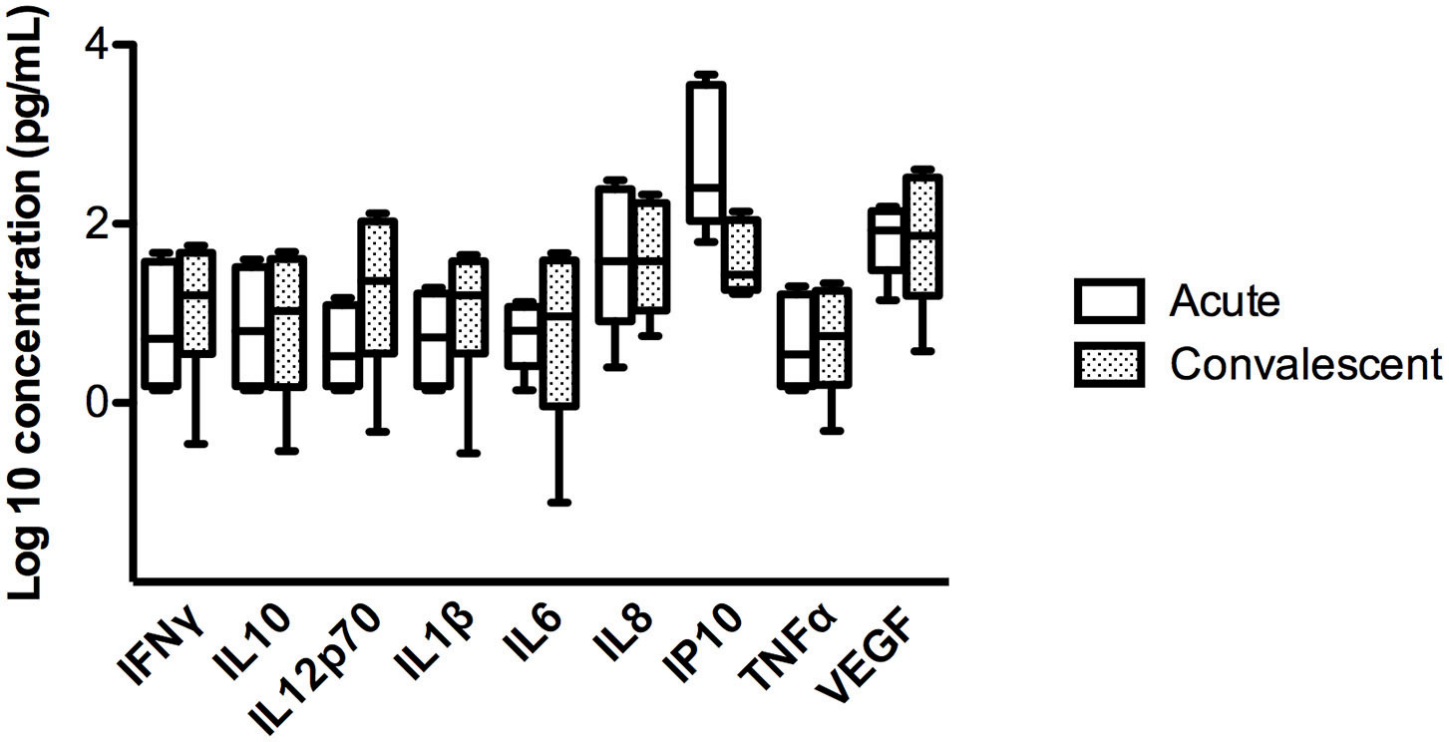
Comparison of concentration of different cytokines in four ANDV-infected cases in acute stage and convalescent stage. Concentrations are represented in pg/mL of INFγ, IL10, IL12p70, IL1β, IL6, IL8, IP10, TNFα, and VEGF. Clear boxes represent values in acute stage and dark boxes values in convalescent stage; whiskers represent minimum and maximum values and horizontal bars in the box the median value (Wilcoxon rank test, *p* < 0.05 = significant).

To assess whether these findings were related to a particular characteristic of the individual or to the acute infection, we studied the concentration of these cytokines in saliva during the convalescence stage (day 60 of the onset of symptoms) in 4 ANDV-infected cases. No significant differences were observed in the concentration of the 9 proteins in the acute and convalescent stage (Figure 2 and Supplementary Table 2).

**Figure 3:**
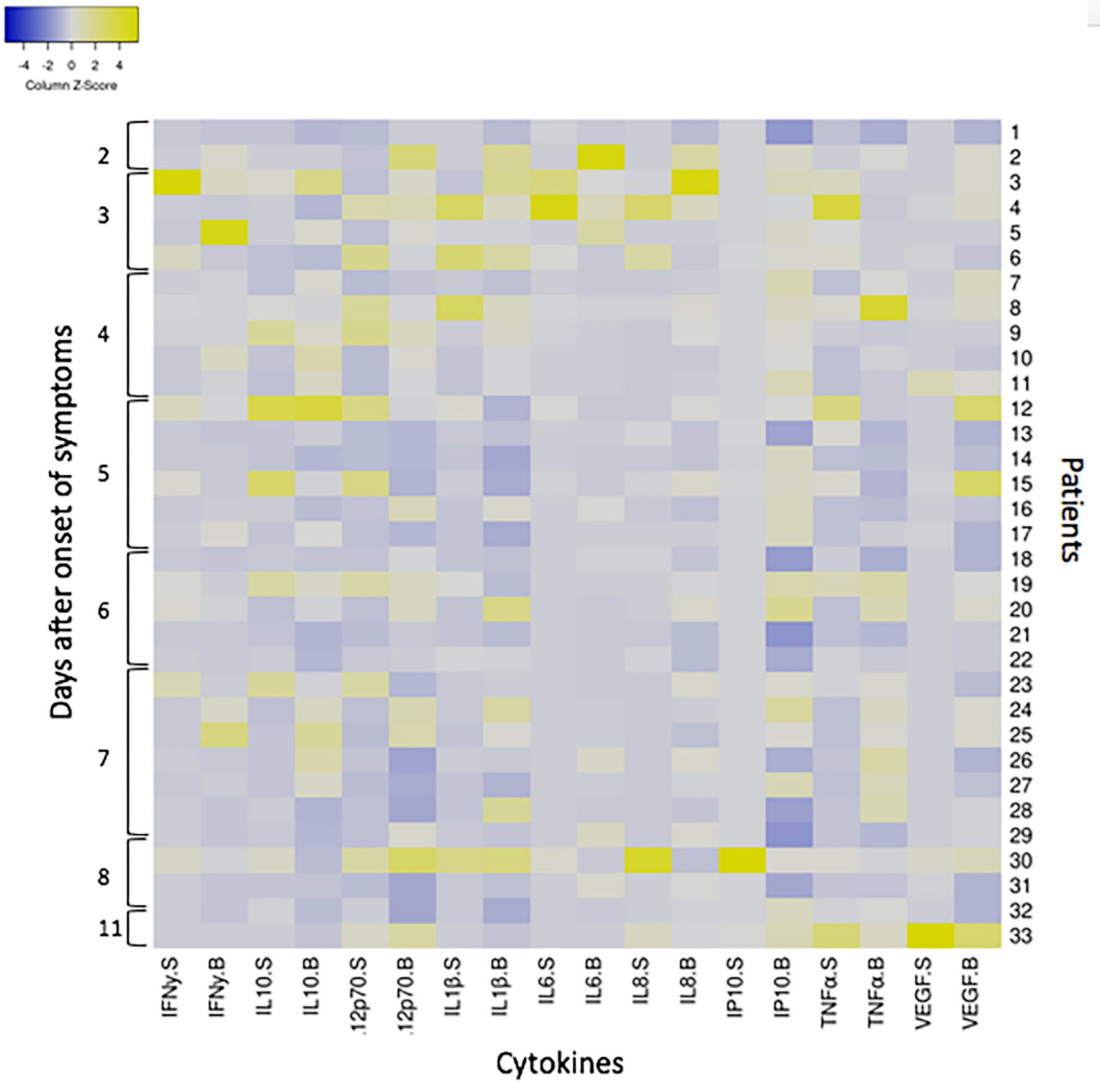
Heatmap showing the cytokine response to ANDV infection in 33 ANDV-infected cases in saliva and serum samples concomitantly taken. The heatmap was constructed in Heatmapper (www.heatmapper.ca; Babicki et al., 2016) according to the fold increase of each cytokine (INFγ, IL10, IL12p70, IL1β, IL6, IL8, IP10, TNFα, and VEGF) concentration of 33 ANDV-infected patients normalized against the median concentration of healthy subjects. Columns represent the Z-scored data for each patient split into two compartments, saliva (S) and serum (B). Rows represents each individual (from 1 to 33) according to days since the onset of symptoms. Colors indicate deviations from the mean of zero (white), as indicated in the color key (Z-score−4 blue, and 4 yellow).

In ANDV infection, an elevated concentration of circulating pro-inflammatory cytokines are observed. In order to evaluate if our findings were related to a local or to a systemic inflammatory response, we compared cytokines concentration in saliva and serum samples obtained concomitantly in 33 ANDV-infected patients during the acute stage of disease. For each individual, a fold increase of each cytokine concentration was calculated compared to median concentration of healthy subjects in saliva and serum samples [median concentration in saliva samples from 38 healthy controls not exposed; median concentration in serum of healthy subjects as described in literature (Kleiner et al., 2013; Angulo et al., 2017; Maleki et al., 2019)]. Results were organized in a heatmap analysis (Figure 3). Interestingly, the greater changes compared to healthy control are seen in the first 4 days after onset of symptoms, in blood and saliva. However, an important variability was observed in the fold increase from each individual. For most all cytokines, no differences in fold increase were observed between saliva and serum concentration. But, there was a tendency of higher increase of IL1β and IL12 p70 in saliva compared to serum, contrary to VEGF and IP10. When we performed a grouped analysis, the differences among fold increase from median concentration of each cytokine in healthy subjects were significantly higher between saliva and serum for all cytokines, except for IL10, IL6, and TNFα (Supplementary Figure 1).

### Description of Mucins in Saliva of Cases and Close Household Contacts

For the analysis of mucins in saliva, the absence/presence of the specific mucin, MUC7, and MU5B (Table 2) was assessed. In addition, the presence of a medium and highly glycosylated isoforms of MUC7 were evaluated (Supplementary Figure 2). The presence or absence of MUC7 in saliva did not show differences between cases and close household contacts (67.7 and 65.3%, respectively) (Table 2). Meanwhile, the analysis of MUC7 isoforms showed significant differences between cases and close household contacts. For close household contacts, there was a predominance of the presence of the 2 isoforms with a frequency of 62.6%, compared to cases, in whom this frequency was 40.5% (*p* = 0.0271). The detection of MUC5B was significantly higher in close household contacts with a frequency of 62.2%, while for cases it was 44.4%, *p* = 0.038 (Table 2).

**Table 2:** Analysis of salivary proteins MUC7 and MUC5B in cases and close household contacts.

	**Mucin**	**Cases *n* (%)**	**Household contacts *n* (%)**	*p*-value^*^
MUC7	Presence	42 (67.7)	49 (65.3)	0.8692
	Absence	21 (33.3)	26 (34.7)	
Isoforms MUC7	Non-glycosylated	8 (19)	2 (4.8)	**0.0271**
	1 Isoform	17 (40.5)	16 (32.6)	
	2 Isoforms	17 (40.5)	31 (62.6)	
MUC5B	Presence	28 (44.4)	46 (62.2)	**0.0381**
	Absence	35 (55.6)	28 (37.8)	

*For the cases, 63 saliva samples were analyzed, while 75 were analyzed for the close household contacts. For the MUC7 isoforms, only the samples with the presence of MUC7 were selected, 42 for the cases and 49 for the close household contacts*.

* *Chi-square test, <0.05 = Significant*.

## Discussion

The present study describes the presence of proteins and immune factors in the saliva of ANDV-infected, their respective close household contacts and healthy controls not exposed to the virus. We found that all cytokines studied were significantly higher in acute cases than in the other two groups. Moreover, close household contacts presented significantly higher concentrations of IFNy, IL12p70, and IL8 in saliva compared to healthy controls not exposed. Concerning salivary IgA, higher concentrations were observed in infected cases than in close household contacts. An inverse phenomenon was observed with the mucins studied, since their presence and different isoforms were significantly higher in close household contacts than in infected cases.

As it has been observed for other viral infections, acutely ill patients in our series showed an increase in salivary IgA, that was not observed in close household contacts (Figure 1B). Although the detection of sIgA was not specific for ANDV, this information suggests that this increase reflects an acute infection. However, it would be interesting to compare this data with the components of saliva of the same patient in a non-diseased condition. In previous studies, we have demonstrated the presence of viral RNA of ANDV in saliva in 16% of the cases during the first week of the onset of symptoms, and of these, 6.2% was able to productively infect VeroE6 cells (unpublished data). On the other hand, Pettersson et al. (2011) showed that the specific sIgA for PUUV has an inverse relationship to the viral RNA levels present in saliva. Since ANDV is the only hantavirus capable of transmitting from person-to-person, it will be important to explore the correlation between the levels of sIgA and viral load of ANDV in saliva. The study of this association as well with the infectivity of ANDV may account for the role of sIgA in saliva and the person-to-person transmission of ANDV.

Regarding the pro-inflammatory immune components detected in saliva, these are significantly increased in ANDV-infected cases. This finding can be related to the acute infection status, but it cannot be ruled out that it is also a condition proper to each individual. This last statement stands in the observation that we did not find any differences in cytokine levels between day 1 (acute) and day 60 (convalescent) in saliva derived from the same patients (Figure 2). However, as we were able to evaluate only 4 patients, it is not yet possible to conclude that this difference between cases and close household contacts may be responsible for greater or lesser susceptibility to acquire the virus.

ANDV-induced illness is associated with the activation of the host's innate immune response. An elevated concentration of circulating TNFα, IFNγ, IL10, and IL6 were observed in ANDV-infected patients compared to healthy subjects (Angulo et al., 2017). Moreover, IL6 levels were associated with more severe symptoms (Angulo et al., 2017; Maleki et al., 2019). Since no differences were observed in fold increase from median concentration of from healthy subjects between saliva and serum samples at individual level, the increase in these cytokines in saliva may be related to a systemic inflammatory response. However, the heatmap analysis (Figure 3) reveals an important inter-individual variability. When we analyzed the results all grouped (Supplementary Figure 1) some cytokines, such as IL1β, IL8, IL12p70, IP10, and VEGF measured in saliva, were highly increased compared to healthy subjects, but lesser in serum. This supports the ability to mount a local immune response. The role that this response may have in the development of the disease still requires more studies. To better understand the cytokine responses in both cases and close household contacts, we included healthy subjects not exposed to ANDV. Interestingly, there were significant differences in IFNy, IL12p70, and IL8 between close household contact and healthy controls not exposed (Figure 1A). These results might suggest a protective reaction against ANDV exposure. Different profiles of cytokines were observed in genital mucosa between HIV infected, HIV exposed but seronegative, and HIV negative individuals (Lajoie et al., 2012). These findings suggest that a specific local response is essential as first line defense during viral exposure. However, these observations are still controversial in HIV-infection. More studies are needed to confirm this hypothesis in ANDV-infections.

Among the proteins included in the study of saliva, mucin highlights for its antiviral properties. Studies conducted *ex-vivo*, have shown that mucins, MUC7 and MUC5B purified from saliva, and MUC1 from breast milk are able to inhibit HIV infection (Habte et al., 2010; Peacocke et al., 2012). On the other hand, assays with viruses pre-treated with 1 mg/mL of mucins decrease the viral titer of HTNV by 30%, while a complete inhibition is observed with concentrations of 25 mg/mL (Hardestam et al., 2008). However, these concentrations are not reported as normal for healthy adults, which vary from 0.1 to 1 mg/mL (Bolscher et al., 1999). Our results show differences in the presence or absence of 2 mucins, MUC5B, and MUC7 (Table 2 and Supplementary Figure 2). MUC5B are characterized by having highly glycosylated tandem repeat domains (Guo et al., 2011). These repetitive sequences create an array of glucan structures that contribute in 50–90% to the weight of the glycoprotein (Bonser and Erle, 2017). MUC5B in rodents is critical for mucociliary clearance and defense function of the airway, and its absence leads to development of chronic bacterial infections, severe inflammation and obstruction of the airway (Welsh et al., 2017). In our results MUC5B presents a higher frequency in the saliva of close household contacts (Table 2 and Supplementary Figure 2). This difference could be a promising marker that could explain, in part, why subjects exposed to the virus did not develop the disease. It would be very interesting in the future to analyze by *ex-vivo* experiments of susceptibility to ANDV infection or viral infection inhibition in presence of purified MUC5B. This could help to understand part of this mechanism and the role of MUC5B in ANDV-infections in humans.

Regarding MUC7, no differences were found in the amount of this mucin, but we found a disparity in the presence of different isoforms. MUC7 is expressed by the submucosal glands and does not constitute part of the mucins that form the mucus. However, it has an antifungal and antibacterial function given its N-terminal end that contains a histatin-type domain (Liu et al., 2000). The isoforms contain an identical composition in amino acids, but with different glycosylation patterns with respect to sialic acid and fucose (Bolscher et al., 1999). These glycosylation patterns could affect the ability of MUC7 to inhibit ANDV infection. To check the status of the mucins at baseline, 4 patients were analyzed, of whom we had acute and convalescent samples and we found no changes between the samples of the same patient (data not shown). Saliva samples from convalescent subjects should be expanded to better explore the role of mucins in susceptibility to ANDV infection.

In summary, through this investigation, significant differences were determined in the oral immune factors between cases and close household contacts. This information should be interpreted with caution given that variations in the content of cytokines and salivary IgA may be modified by the acute disease condition of the cases. However, our findings propose new fields to study and contribute to the understanding of host susceptibility in this infectious disease. The role of mucins and their isoforms in the inhibition of ANDV infection seems to be a promising investigation area to expand. The importance of describing these factors in this population will be very useful to clarify the mechanisms of acquisition of ANDV, particularly in person-to-person transmission of ANDV.

## Data Availability Statement

The raw data supporting the conclusions of this article will be made available by the authors, without undue reservation.

## Ethics Statement

The studies involving human participants were reviewed and approved by Ethics Committee of the Faculty of Medicine. Pontificia Universidad Católica de Chile (Code 12-292 and 16-092). All participants signed an informed consent at the time of enrollment, this consent was approved by the same committee. Written informed consent to participate in this study was provided by the participants' legal guardian/next of kin.

## Author Contributions

MF, CM-V, JA, and NL designed the study. CA and CM-V performed the experiments. NL, CM-V, and JA analyzed the data. CM-V, JA, MF, and NL wrote the paper. CH performed control subject's enrolment, organization of data collection, and basic statistical analysis. All authors contributed to the article and approved the submitted version.

## Conflict of Interest

The authors declare that the research was conducted in the absence of any commercial or financial relationships that could be construed as a potential conflict of interest.
