# Supplementary material for: Characterization of Oral Immunity in Cases and Close Household Contacts Exposed to Andes Orthohantavirus (ANDV)
Source: Front Cell Infect Microbiol. 2020 Nov 3;10:557273. doi: 10.3389/fcimb.2020.557273 (PMC7670062; doi:10.3389/fcimb.2020.557273)
Supplement: Supplementary file 1 [file Data_Sheet_1.docx]

**SUPPLEMENTARY RESULTS:**

**Table S1:** Summary of the measurement of cytokines (pg/mL) and sIgA (mg/mL) in saliva of ANDV-infected cases, close household contacts and healthy controls not exposed.

|  | **ANDV-infected cases**  **n=69** | |  | | **Close household contacts**  **n=76** | | | **Healthy not exposed**  **n=39** | | |  | |
| --- | --- | --- | --- | --- | --- | --- | --- | --- | --- | --- | --- | --- |
| **Factor** | **N /total (%)** | **Median** | | **N/total (%)** | | **Median** | **N/total** | | **Median** | **p value*** | |  |
| IFNγ | 49 (71) | 5,19 | | 71 (93) | | 2,95 | 39 | | 1,42 | **<0.0001^a^** | |  |
| IL10 | 48 (70) | 4,79 | | 70 (92) | | 2,91 | 39 | | 2,06 | **0.0105^b^** | |  |
| IL12p70 | 49 (71) | 6,02 | | 71 (93) | | 3,34 | 39 | | 1,65 | **<0.0001^a^** | |  |
| IL1ß | 49 (71) | 12,89 | | 70 (92) | | 5,03 | 39 | | 2,70 | **0.0015^c^** | |  |
| IL6 | 49 (71) | 6,12 | | 70 (92) | | 3,19 | 39 | | 2,06 | **<0.0001^b^** | |  |
| IL8 | 49 (71) | 116,5 | | 70 (92) | | 28,5 | 39 | | 8,49 | **<0.0001^a^** | |  |
| IP10 | 49 (71) | 803 | | 70 (92) | | 34 | 39 | | 28,51 | **<0.0001^b^** | |  |
| TNF**α** | 49 (71) | 7,78 | | 69 (91) | | 3,14 | 39 | | 2,06 | **0.0098^c^** | |  |
| VEGF | 49 (71) | 92 | | 71 (93) | | 33 | 39 | | 41,67 | **0.00011^d^** | |  |
| sIgA | 66 (96) | 0,27 | | 71 (93) | | 0,17 | 39 | | **ND** | **0.0271** | |  |

*Kruskal-Wallis test and Mann Whitney test, p<0.05=significant

Comparisons are significance between columns:

a: all group are statistically significant

b: all group are statistically significant, except for Healthy controls not exposed and Close household contact

c: only between Cases and Healthy controls not exposed is statistically significant

d: only between Cases and Close household contact is statistically significant.

**Table S2:** Comparison of concentration (pg/mL) of different cytokines in 4 ANDV-infected cases in acute stage (Day 1; D1) and convalescent stage (Day 60; D60).

|  | **Median concentration**  **(pg/mL)** | | |
| --- | --- | --- | --- |
|  | **D1** | **D60** | **p value** |
| IFNγ | 5.2 | 16.03 | 0.875 |
| IL10 | 6.36 | 10.68 | 0.875 |
| IL12p70 | 3.32 | 23.01 | 0.625 |
| IL1β | 5.38 | 15.96 | 0.625 |
| IL6 | 6.46 | 9.25 | 0.875 |
| IL8 | 38.35 | 38.2 | 0.875 |
| IP10 | 252 | 27.02 | 0.125 |
| TNF**α** | 3.49 | 5.6 | 0.875 |
| VEGF | 85 | 73.6 | 0.875 |

Wilcoxon rank test, p<0.05=significant

LEGEND OF SUPPLEMENTARY FIGURES:

**Figure S1: Fold increase concentration of cytokines in 33 ANDV-infected cases compared to healthy subjects in saliva and serum samples.** Fold increase was calculated as a ratio of each cytokine concentration from patients and the median concentration of each cytokine from healthy subjects in saliva and serum sample. For both, patients and healthy subjects, concentrations are expressed in pg/mL. Clear boxes represent fold increase for saliva and dark boxes for serum; whiskers represent minimum and maximum values and horizontal bars in the box the median value. Dotted line represents a fold increase of 1. **p<0.005, ***p<0.0005 and ****p<0.0001 (Wilcoxon rank test, p<0.05=significant).

**Figure S2:** **Analysis of the presence/absence of Muc7 and Muc5B by Western Blotting.** Representative western blotting used to show the different migration across de SDS-PAGE between mucins isoforms. **A**) Detection of MUC7 isoforms, lanes 1-9 correspond to saliva of ANDV-infected cases, L correspond to protein ladder and each band represent differences in the glycosylation profiles. The bands that run further have less glycosylations. **B)** Detection of MUC5B, lanes 1-9 correspond to saliva of ANDV-infected cases, and L correspond to protein ladder. In both gels, 20 µg of total protein was loaded per well. The ladder was overexposed for better visualization.
