# Supplementary figures and images for: Characterization of Oral Immunity in Cases and Close Household Contacts Exposed to Andes Orthohantavirus (ANDV)
Source: Front Cell Infect Microbiol. 2020 Nov 3;10:557273. doi: 10.3389/fcimb.2020.557273 (PMC7670062; doi:10.3389/fcimb.2020.557273)

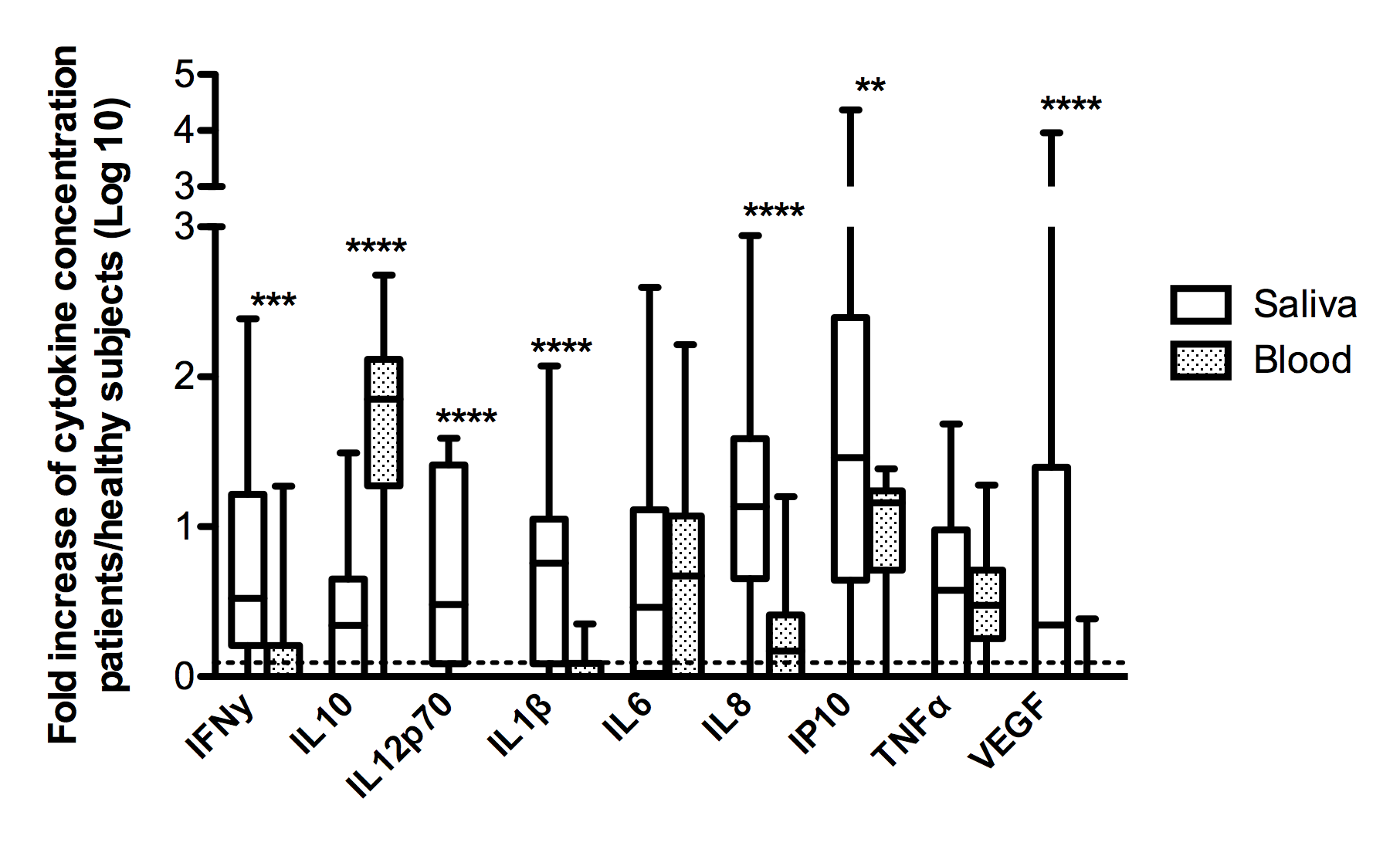

Supplement: Supplementary file 2 [file Image_1.TIFF]

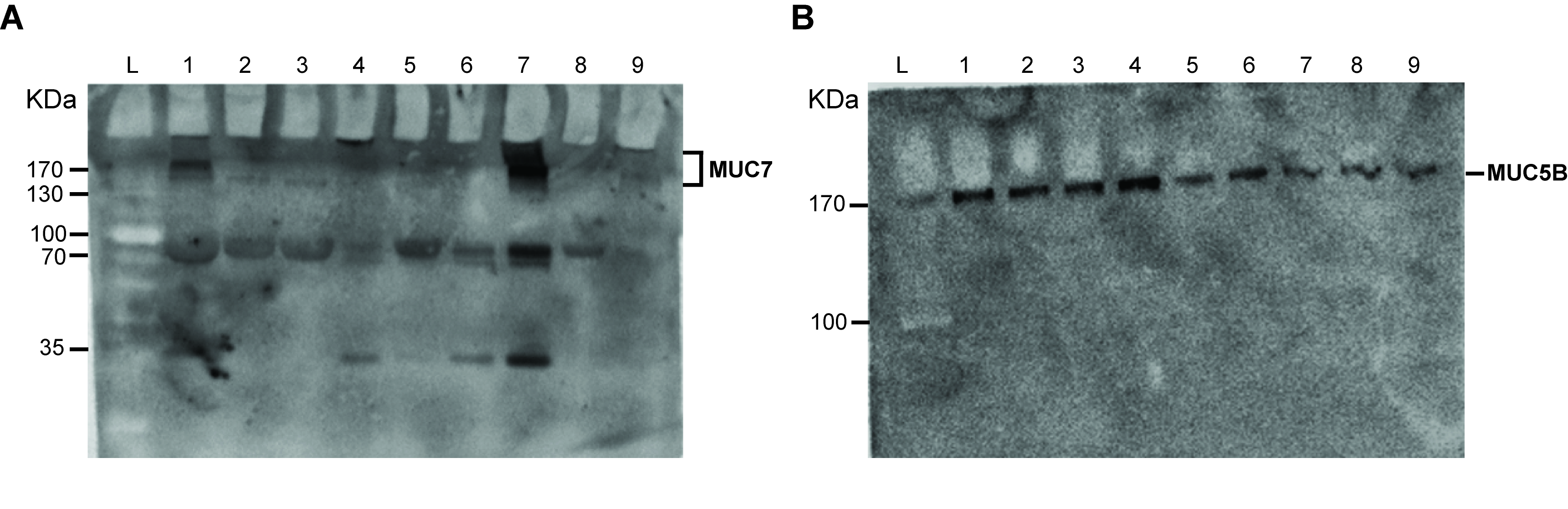

Supplement: Supplementary file 3 [file Image_2.TIF]
